# Tracking the Migration of Injectable Microdevices in the Rodent Brain Using a 9.4T Magnetic Resonance Imaging Scanner

**DOI:** 10.3389/fnins.2021.738589

**Published:** 2021-10-05

**Authors:** Adam Khalifa, Jonah Weigand-Whittier, Christian T. Farrar, Sydney Cash

**Affiliations:** ^1^Department of Neurology, Massachusetts General Hospital, Harvard Medical School, Boston, MA, United States; ^2^Department of Radiology, Massachusetts General Hospital, Athinoula A. Martinos Center for Biomedical Imaging, Harvard Medical School, Boston, MA, United States

**Keywords:** MRI, wireless, migration, central nervous system, injection

## Abstract

Wirelessly powered microdevices are being miniaturized to improve safety, longevity, and spatial resolution in a wide range of biomedical applications. Some wireless microdevices have reached a point where they can be injected whole into the central nervous system. However, the state-of-the-art floating microdevices have not yet been tested in chronic brain applications, and there is a growing concern that the implants might migrate through neural tissue over time. Using a 9.4T MRI scanner, we attempt to address the migration question by tracking ultra-small devices injected in different areas of the brain (cortico-subcortical) of rats over 5 months. We demonstrate that injectable microdevices smaller than 0.01 mm^3^ remain anchored in the brain at the targeted injection site over this time period. Based on CD68 (microglia) and GFAP (astrocytes) immunoreactivity to the microdevice, we hypothesize that glial scar formation is preventing the migration of chronically implanted microdevices in the brain over time.

## Introduction

**FIGURE 1:**
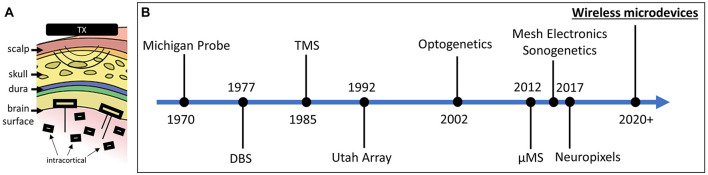
**(A)** Two types of free-floating microdevices: epicortical with penetrating electrodes and intracortical. **(B)** Timeline showing the significant breakthroughs in the field of neural interface (interface modalities and technologies are both shown).

Fully injectable wirelessly powered devices, or microdevices, play a major role in many emerging biomedical applications including neural monitoring (Lee et al., 2020; Sigurdsson et al., 2020; Zhang et al., 2020), neural stimulation (Freeman et al., 2017; Khalifa et al., 2019), and temperature sensing (Cortese et al., 2020; Shi et al., 2021). Microdevices in this case should not be confused with micro/nanoparticles (Chen et al., 2018; Le et al., 2019; Kozielski et al., 2021) as the latter does not include integrated circuitry. Wireless microdevices for brain interface (recording and stimulation) can be placed into two categories: epicortical and intracortical (Figure 1A). Both types require a transmitting (Tx) external system that is responsible for transmitting power and data to the implanted device. Intracortical microdevices offer more flexibility compared with epicortical microdevices, as they can be directly injected anywhere into the brain, but are more challenging to microfabricate and power due to their smaller footprint. Although only a handful of academic research labs have managed to microfabricate brain devices small enough to be fully injectable, as researchers continue to make progress in reducing the microdevice volume using innovative wireless powering (Karimi et al., 2017) and packaging techniques (Khalifa et al., 2017a), future developments will no doubt result in more epicortical implants transform into intracortical implants (Barbruni et al., 2020; Yang et al., 2020).

The advantages of using wireless intracortical microdevices are numerous. When injected into the central nervous system (CNS) and when made individually addressable, stand-alone microdevices can be scaled and used to interface with hundreds of neurons in a distributed fashion (Dinis and Mendes, 2020). Clinicians and researchers would benefit greatly from using microdevices because of their ability to create different electrode configurations for distributed animal or human neural interfaces. One of the biggest strengths of wireless microdevices is their immunity to micromotion. It has been shown in many research studies that floating implants that are not otherwise anchored to the skull or in contact with meningeal tissue mitigate injury responses (Biran et al., 2007; Kim et al., 2013; Ersen et al., 2015). Lastly, intracortical microdevices are compatible with minimally invasive intracranial insertion techniques (Cortese et al., 2020; Sigurdsson et al., 2020; Khalifa et al., 2021), which leads to reduced surgical complications and faster recovery. Unlike bulky implantable electronics, injectable microdevices require only minimally invasive superficial surgeries. Our group (Khalifa et al., 2021) and others (Zhou et al., 2017) have shown that a small immune response was caused when inserting a syringe into the brain.

Since the microdevice technology is still in its infancy, to the best of our knowledge, chronic animal experiments (>1 month) with fully injectable microdevices have not yet been conducted. For these novel devices to perform their function properly, they must be precisely injected into the correct region. It is then crucial for the successful operation of floating microdevices to remain in the targeted sites. This leads to the question as to whether the stability of the location of injected microdevices relative to the neural tissue carries over to chronic *in vivo* experiments. More specifically, our study aims at answering the following questions: (1) Will intracortical microdevices (≤0.01 mm^3^) drift by more than 200 μm within a period of 4.5 months? (2) Does the site of injection and size of the microbead impact migration or anchoring? (3) What could be causing migration or anchoring? 200 μm is chosen as the lower limit due to the resolution limit of the MRI scanner used in this study.

The question of whether floating microdevices will migrate is not trivial given the small scale of the device and the lack of proper anchor points within the tissue. Numerous factors could cause migration such as pulsing of blood in vessels, breathing, or simply locomotion. When the heart pumps blood into the brain, the brain expands and contracts due to periodic variations in arterial blood pressure. Gilletti and Muthuswamy (2006) have shown that, in anesthetized rats, pulsatile surface micromotion was observed to be in the order of 10–30 μm due to pressure changes during respiration and 2–4 μm due to vascular pulsatility. The immune response could also play a major role. Due to the mechanical mismatch and insertion damage, it is expected that the immune system will create an inflammatory response and sheath of astrocytes between the microdevice and the neural tissue that could move the implant away from the target placement area.

Microdevice migration will have severe consequences for moving these devices into a translational role in the clinical domain. Most clinical purposes and targeting require a high degree of accuracy and precision when small neuronal volumes are targeted for treatment. Device migration would alter the neuronal population activated or monitored by the device. A direct comparison between electrode localization and clinical outcomes has been shown in multiple studies. For instance, research shows that DBS electrode migration (>2 mm) due to traumatic brain injury will lead to poor clinical outcomes (Young et al., 2011) and that DBS electrode displacement due to subdural air invasion leads to a decrease in stimulation effects in patients suffering from Parkinson’s disease (Van Den Munckhof et al., 2010).

If microdevices are indeed stationary following implantation, then the number of applications and possible clinical therapeutic uses would expand significantly, possibly becoming a next-generation type of intracortical electrode used for diagnosis and treatment (Figure 1B). On the other hand, if microdevices are found to drift over time, then any potential displacement after the injection must be factored into the expectations from what can be achieved with this new technology.

## Materials and Methods

### Injected Device

**FIGURE 2:**
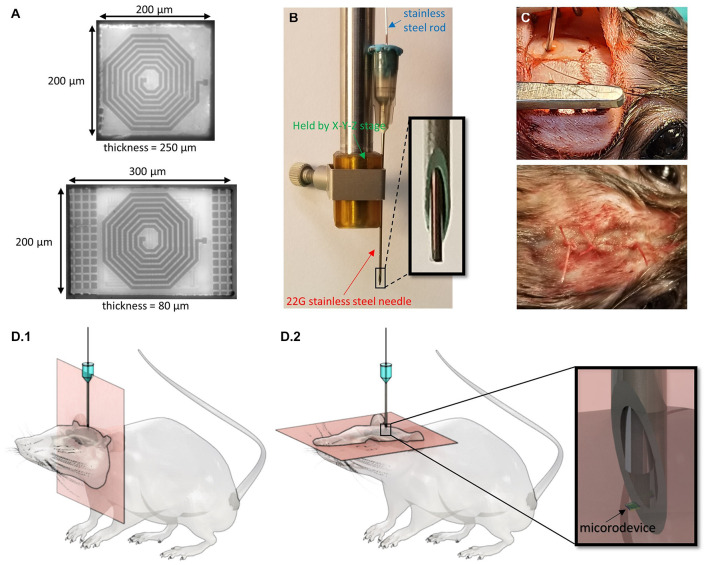
Techniques used to inject the microdevices and track their location. **(A)** Micrographs of two microdevices (D1 is bottom and D2 is top) of different thicknesses and surface areas. **(B)** A picture of the injection tool with a close-up of the 22G needle and rod tip. **(C)** Picture of the rat during microdevice injection and after the skin is sutured. **(D)** 3D drawings of the rat with an injected microdevice in the brain. MR images are taken using (D.1) coronal orientation and (D.2) axial orientation.

We used microdevices with two different dimensions: 300 × 200 × 80 μm (referred to as D1) and 200 × 200 × 250 μm (D2) (Figure 2A). These devices represent two different versions (the smallest and largest) of the “microbead,” a functional ultra-small stimulating device powered by inductive coupling. The content of the implant is of no importance for the study and thus will not be described, but more details can be found in Khalifa et al. (2017b, 2019). The microdevices were not powered during the study as there is no reason to believe that transmitting RF energy while respecting the specific absorption limit would have affected their migration or anchoring. Relevant to this migration study are the microdevice’s dimensions, shape, and properties of its encapsulation coating. The implants are shaped by dicing and grinding an application-specific integrated circuit (ASIC) die. The microdevices are then coated with a 5-μm thick SU-8 layer (Kayakua, MA, United States) that was inkjet printed using a Dimatix Fujifilm DMP 2800 printer. SU-8 material was chosen for its chronic biocompatibility as it does not show apparent signs of tissue damage or inflammatory reaction over many months *in vivo* (Cho et al., 2013; Márton et al., 2020).

### Injection Method and Surgical Procedure

The microdevices were injected using a custom-made novel injection tool described in detail in Khalifa et al. (2021). The main components of the injection tool are a 22G (or thin wall 23G) needle, and 350 μm stainless steel cylindrical rod (OrthoTechnology, FL, United States; Figure 2B). The needle is used to facilitate dura penetration. A biocompatible and bio-dissolvable material (polyethylene glycol) is used to temporarily affix the microdevice to the tip of the rod. To pick up and inject the microdevice, we mounted the needle on a stereotactic fixture to an X-Y-Z micro-positioner that allows for accurate and controlled positioning of the injection tool.

All research protocols were approved and monitored by the Massachusetts General Hospital (MGH) Institutional Animal Care and Use Committee (IACUC). Five male Brown Norway rats (340–380 g, Charles River Labs, MA, United States) were used in this study. All the adult rats were older than 8 months, as it was important to avoid the brain from increasing in volume during the course of this study. The rat was anesthetized with 2–3% isoflurane throughout the surgery. After shaving the hair over the surgical site, the animal was immobilized on a stereotaxic frame, and the scalp was disinfected and numbed with lidocaine. A heating pad was placed below the animal to maintain body temperature. Up to 1.5 cm sagittal incision was made in the skin over the skull, and burr holes were drilled using a precision surgery dental drill with a 0.79 mm carbide bur. Two to four 0.8 mm diameter burr holes were drilled in the skull anywhere between −5 and 3 mm anterior and between ±2.5 and ±3 mm lateral to the bregma. The holes were rinsed with sterile PBS to remove drill debris and blood. It was crucial to clean the skull as much as possible as blood is rendered dark in T2-weighted imaging and could thus make it difficult to spot the microdevices. The needle, the rod, and the microdevices were sterilized using ethylene oxide solution and rinsed with DI water. The pick-and-inject method described above was then used for the injection by carefully positioning the needle right over the burr hole. The needle tip punctured the dura and reaches the desired implantation depth which could be anywhere between −1.5 and −7 mm DV (Figure 2C). Multiple brain regions were targeted such as the primary motor cortex, the hippocampus, the striatum, the corpus callosum, and the thalamus. For some of the rats, instead of injecting a microdevice, we implanted a tungsten microelectrode (TM33B10, World Precision Instruments, Bedford, NH, United States), to compare the immunoreactivity of the injected microdevice to that of a tethered microelectrode measuring 256 μm in diameter. The microelectrode was anchored to the skull by covering the burr hole with dental cement. The burr holes that were used to inject the microdevices were covered with bone wax. The skin was then sutured shut using 4-0 absorbable polyglactin 910 (Vicryl coated) sutures. In addition, tissue adhesive (VetBond by 3M) was applied to reinforce the wound closure (Figure 2C).

### MRI Setup and Settings

To track the microdevices after injecting them into the brain, their location was localized directly on MR images and then again after brain extraction and trimming. The rat was brought to a 9.4T MRI scanner (Biospec 94/20, Bruker Biospin, Billerica, MA, United States). Before beginning the MRI scan, the rat was placed on a cradle within the MRI scanner. During the MRI scan, the rat was sedated with an oxygen/room air/isoflurane mixture, which was delivered by a nose cone placed over the snout of the rat. The nose cone was firmly attached to the cradle on which the rat lies, keeping it in place. The rat was secured in the cradle using standard hospital adhesive tape. A 1H quadrature volume coil (Bruker Biospin) was used for transmission, while a ^1^H 4-channel phased array coil was used for reception with the phased array coil placed over the head. Imaging was done using a T2-weighted turbo spin-echo (TSE) RARE (Rapid Acquisition with Relaxation Enhancement) sequence. The following parameters were used: TR/TE = 4011 ms/36 ms, RARE factor 8, 15 signal averages, FOV = 3 cm^2^, matrix size = 240 × 240, and 35 slices with a 0.25 mm slice thickness. Therefore, the voxel dimensions are 125 × 125 × 250 μm. The signal-to-noise (SNR) ratio and resolution were chosen such that the microdevice size on the MR images showed a margin of error of less than 200 μm. The scanning lasted ∼30 min. In an attempt to increase SNR and resolution, some of the MRI sequences included longer scanning time (50 min) or smaller slice thicknesses (0.15 mm). MR images were taken using coronal and axial directions (Figure 2D). During the scanning, the rat was kept warm using hot air. The respiration rate was monitored throughout the imaging session. At the end of the scan, the rat was removed from the scanner and returned to its cage.

**FIGURE 3:**
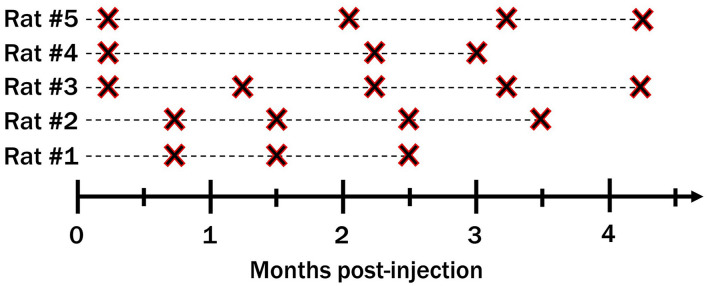
Timeline of MRI scans taken for all five rats.

Figure 3 shows the timeline of MR scans for all five rats. The rats were euthanized shortly after their last scan. The number of MR scans taken and the timing between the scans varied for different rats. Some were scanned five times and up to 17 weeks post-injection. Longer post-injection times were not investigated as the microdevice were injected into adult rats (>8 months) which typically have a lifespan of 24 months, but is much shorter for the rats we worked with since they underwent invasive brain surgery. Additional information such as the number of microdevices injected for each animal, and their location in the brain can be found in Supplementary Figure 1.

### Image Analysis

After data collection, the images are analyzed using the Freeview tool in the FreeSurfer software. MR images from different scans taken at different times are superimposed to look for any possible migration. This was done by first aligning the images using easily identifiable markers (the skull being the main marker). Alignment was done by adjusting the transform parameters (X, Y, Z for translation and rotation). Microdevices are then spotted by finding the injection 3D coordinates (AP, ML, and DV) or by locating the cortex dimple created by the injection tool. Microdevices are rendered dark in T2-weighted imaging. Finally, all images except one were set to 50% opacity. If a drift was noticed, then the center-to-center distance of the microdevices was measured. Images also underwent subtraction instead of superimposition which allowed us to more easily spot migrations that would be indicated by bright pixels. Drifts on the Z-axis are directly spotted using coronal slices, but can also be spotted using multiple axial slices which could be used to reconstruct a 3D image of the brain.

### Tissue Preparation and Histological Analysis of Brain Reactivity

In an attempt to answer the question of what is causing the migration or anchoring, we decided to investigate the immunoreactivity around the microdevices. It involved staining for the expression of markers associated with macrophages, cluster of differentiation 68 (CD68), and astrocytes, glial fibrillary acidic protein (GFAP). At designated time points, the animals were euthanized with a solution of pentobarbital and perfused. The brain was then extracted, immersion-fixed in PFA, and trimmed down to a small block of tissue. We decided not to remove the implant before the histological preparation so as to not cause a detrimental impact on the integrity of the true implant/tissue boundary. On the other hand, if a microelectrode was implanted into the brain, then it had to be carefully removed as sectioning with the electrode would have led to distorted or stretched slices. The tissue block was dehydrated through graded alcohol and xylene and embedded in paraffin. Some of the paraffin-embedded tissue blocks were sectioned (4 μm) parallel to the injection track (coronal slices), while other blocks were sectioned perpendicular to the injection track (axial slices). The sections were incubated with primary antibodies anti-GFAP (ab7260, Abcam, 1:100 dilution) and anti-CD68 (ab201340, Abcam, 1:100 dilution) at 4°C overnight. The sections were then incubated in secondary antibodies, goat anti-rabbit Alexa Fluor 488 (ab150077, Abcam, 1:400 dilution), and goat anti-mouse Alexa Fluor 555 (ab150118, Abcam, 1:400 dilution) for 2 h in the dark at room temperature. Finally, the slides were stored in the dark at 4°C until images were collected using a Nikon epifluorescence inverted microscope at 20×. We chose axial and coronal sections that were as close as possible to the microdevice (less than 4 μm away) to capture regions with the largest tissue response.

## Results

**FIGURE 4:**
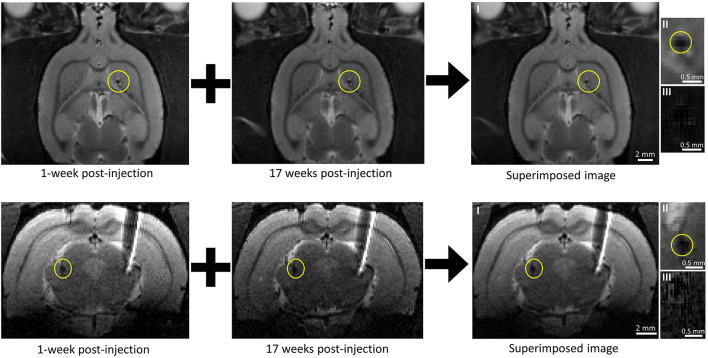
Tracking microdevice migration in the rodent brain using a 9.4T MRI scanner. (*Top row*) Two axial MR images of the rat (#3) brain taken at different post-injection times and one axial MR image created by superimposing one over the other. The microdevice was injected (encircled in yellow) in the right hemisphere. (*Bottom row*) Two coronal MR images of the rat (#5) brain taken at different post-injection times and one axial MR image created by superimposing one over the other. The microdevice was injected (encircled in yellow) in the left hemisphere. The right hemisphere shows a tethered microelectrode. Images II show a close-up of the microdevice taken from images I, and images III show a close-up view of the microdevice with subtraction done instead of superimposition.

We have previously shown that the spatial precision of our injection method was excellent as the microdevice reached within 200 μm of its target location 91% of the time (Khalifa et al., 2021). In this study, all 11 microdevices in all five rats reached their target site. The microdevices exhibited good compatibility with MRI with few artifacts and noise (Figure 4). In most MR images, the microdevices measured about 250 to 400 μm, which was close to the actual microdevice size (200 μm for D2 and 300 μm for D1). We did not observe any microdevice migration in any of the rats. Among the 21 MR scans taken using the 9.4T MRI scanner, we show a subset of four MR images (Figure 4), two from rat #3 and two from rat #5 as they had the longest post-injection times. Figure 4 compares an MR image 1-week post-injection with one taken at 17 weeks post-injection. For rat #3, the observed microdevice (D2) was injected into the striatum. As for rat #5, the encircled microdevice (D2) was injected into the thalamus. Examples of MR images showing microdevices that were injected into M1 and the corpus callosum are shown in Supplementary Figure 2. Rat #5 also had a tungsten microelectrode implanted into the right hemisphere, but the electrode appears much larger than its actual size due to the large susceptibility artifact. After euthanizing the rats, we confirmed the location of microdevices by extracting the brain and carefully trimming it (Supplementary Figure 3).

MR images taken with other sequences chosen to further increase SNR and resolution were not successful (Supplementary Figure 4) as they showed severe artifacts. Gradient duty cycle issues occurred after decreasing the slice thickness down to 0.15 mm, and the motion artifact often appeared after increasing the scan time up to 50 min.

**FIGURE 5:**
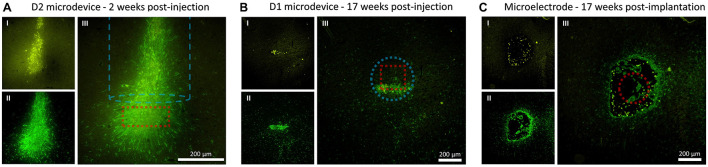
Fluorescence microscopy images showing CD68 (microglia) and GFAP (astrocytes) immunoreactivity to the microdevice and microelectrode. **(A)** Coronal tissue slice at 2 weeks microdevice post-injection. **(B)** Axial tissue slice from rat #5 at 17 weeks microdevice post-injection. **(C)** Axial tissue slice from rat #5 at 17 weeks microelectrode post-implantation. Images I show CD68 (yellow), II show GFAP (green), and III merge I and II. The dashed red line shows the estimated location of the microdevice or microelectrode. The dashed blue line shows the injection track and outlines the dimensions of the injection tool.

In order to evaluate the inflammatory tissue response and damage associated with the implantation, the amount and distribution of microglia and astrocytes were visualized. Figure 5 shows the microphotographs of the neocortex double stained with antibodies for GFAP and CD68. Figure 5A shows a section cut parallel to the injection track and therefore also includes the immunoreactivity to the injection tool (the dashed blue line outlines the injected rod). Figure 5B shows a section cut perpendicular to the injection track. At 17 weeks post-injection, the hole created by the injection tool was almost entirely closed and healed; therefore, there was almost no astrocyte and microglia accumulation near the injection track which then allowed us to better detect the astrocyte and microglia surrounding the microbead. Since the microbead was not perfectly rotationally aligned to the section, only one of its 12 edges was in close proximity to the extracted slice (<4 μm). This explained why only a strip of astrocyte and microglia was observed. Figure 5C shows a section cut perpendicular to the implanted microelectrode.

## Discussion

If floating wireless microdevices are to become a major technology for interfacing with the central nervous system, then we can no longer ignore the important open question as to whether the spatial stability of the microdevices after injection can be preserved under chronic conditions. This research aims to investigate this unexplored area.

MRI was used to find the three-dimensional coordinates of the chronically implanted microdevice and to track its migration over time in rats. At first, MRI did not seem like the optimal imaging technique for this research due to compatibility issues between large magnetic fields and most metallic objects. For instance, some patients with implanted electrodes or devices are not admitted to MRI scanners due to safety hazards. The electromagnetic (EM) field generated during MRI can induce electromotive forces on metallic objects which will increase their temperature and the possibility of damaging the surrounding tissue. In addition to safety concerns, electrodes often generate imaging artifacts due to their large size and the difference in magnetic susceptibility between the metal and the surrounding tissue, hindering accurate visualization around the electrode (Serano et al., 2015). Fortunately, all these issues are eliminated with microdevices that are mainly composed of an ASIC and microelectrodes. Although most materials that conduct current will induce a very large susceptibility artifact in the MR system, the microdevices showed minimal susceptibility artifacts (<100 μm). As can be observed in Figure 4, the tungsten microelectrode (TM33B10, World Precision Instruments, Bedford, NH, United States) implanted into the right hemisphere shows a much larger susceptibility artifact (>1 mm) although its diameter is smaller than that of the microdevice. There are three main explanations for this: (1) the most abundant metal used in the silicon chip is copper, which is not a ferromagnetic material; (2) all metallization represents less than 2% of the entire chip, which is mainly composed of silicon and silicon dioxide; (3) the integrated 2D electrodes are composed of a very thin layer of a non-ferromagnetic material. These reasons also explain why the exertion of attractive or rotational forces onto the microdevices is negligible and therefore not capable of moving the implant during imaging.

Although MRI has shown to be a great imaging tool for this study, it does also have limitations on its ability to track ultra-small devices. For instance, even with a strong magnet (9.4T), displacements smaller than 200 μm become difficult to measure in our MR images due to the limited SNR and resolution. These limitations also made it very difficult to know if the microdevices have rotated at some point throughout the study. One way to increase SNR was by increasing the number of signal averages (NSA) which comes at a cost of increased scan time. However, scan times over 30 min increased the chances of motion artifacts. We hypothesized that isoflurane anesthesia caused erratic respiration which caused blurring in the images, making it difficult to track the microdevice. In order to increase the number of slices (which increased SNR and spatial resolution), we have also attempted to use smaller slice thicknesses. However, due to gradient duty cycle issues, this could not be accomplished without significantly increasing the repetition time and hence scan time. We have also attempted to track much smaller wireless microdevices called optical wireless integrated circuits (OWICs). The OWICs were fabricated by a laboratory in Cornell University and measured only 200 × 75 × 4 μm (Cortese et al., 2020). We were not able to spot these ultra-thin (4 μm) microdevices due to the much larger slice thickness (250 μm) of our sequence.

Nevertheless, the scanned images were able to show that intracortical microdevices (≤0.01 mm^3^) do not drift by more than 200 μm within a period of 4.5 months. To investigate whether different brain regions impacted the result of this study, we injected the microdevices into six brain regions: the primary motor cortex, the hippocampus, the stratum, the corpus callosum, and the thalamus. We chose these regions for two reasons: (i) they are common neural interfacing areas to treat mental and neurological disorders, and (ii) their tissue properties are different. To investigate whether the size of the microdevice had any effect on its migration, we used two microdevices of different volumes with D2 (0.01 mm^3^) volume being more than twice that of D1 (0.0048 mm^3^). Neither the brain region nor the microdevice volume has had any effect on the results as migration of more than 200 μm has never been observed during our study.

In an attempt to better understand the possible anchoring effect, the immunoreactivity around the microdevice was investigated. Reducing the biological responses in the brain parenchyma is one of the many advantages of using a floating microdevice. As effort is being made to decrease the size of microdevices, we would soon have microdevices that have similar diameters as penetrating microelectrodes (<100 μm). However, microdevices are currently not small enough to be “invisible” to the immune system. Immunohistological data show astrocytes and microglia formation around the microdevice, which suggests that the ultra-small device coated with SU-8 material elicits typical immune responses, even after 17 weeks post-injection. Interestingly, the spatial extent of GFAP and CD68 around the injected D1 microdevice was comparable to that surrounding the tethered tungsten microelectrode (Figure 5 and Supplementary Figure 5). The only noticeable major difference was that the tungsten microelectrode caused a large tissue opening along the entire electrode track, whereas the tissue above the microdevice was allowed to close and heal. We hypothesized that scar formation, unwanted in most applications since it degrades long-term interface with the neural system, was helping anchor the microdevice in the target site. Therefore, an interesting question is whether further reducing the scar formation could trigger the migration of free-floating microdevices in the brain, and if so, then what is the minimum amount of glial encapsulation required to prevent migration? It is important to remember that if the microdevices were smaller and used a superior chronic encapsulation layer, the immune response would be activated during injection since damaging the blood–brain barrier (BBB) was inevitable due to the vast capillary beds that span the cortex. Furthermore, the shown immunoreactivity around the microdevice was specific to the ones used in this study. If another type of wireless microdevice is to be used, then migration could still be a possibility. The biological response to any implanted medical device is a function of the size and shape of the device, the properties of the encapsulating materials, the injection method used, and the skill of the surgeon (Anderson, 2001).

Lastly, as wireless microdevices are mainly designed for human applications, an interesting question that is beyond the scope of our current experimental study is whether the findings of this research would also apply to the human brain. At the scale of the human brain, cardiac and respiratory pulsations are significantly more profound, which can be easily observed in Terem et al. (2018), where the extent of brain motion in human subjects has been captured using a 3T amplified magnetic resonance imaging. Furthermore, apart from the fact that the rat brain volume is approximately 0.1% that of the human, the Young’s modulus for the human cortex is around 0.6–15.2 kPa (Green et al., 2008), whereas for the rat cortex, it is down to 0.03–1.8 kPa (Elkin et al., 2010). Although all these differences could make it difficult to predict how the tissue will interact with microdevices in the human brain, the results of this study provide a good starting point for discussion and further research.

## Conclusion

One of the biggest advantages of wirelessly powered microdevices over tethered electrodes is that they have the potential of moving with the surrounding tissue and therefore can remain close to the targeted group of neurons. However, this advantage has never been confirmed for chronically implanted microdevices. This study investigates whether the migration of stereotactically implanted microdevices in rats could occur in the weeks or months following surgery. Our study shows that microdevices smaller than 0.01 mm^3^ remain anchored in the rat brain at the targeted injection site. The findings of this research show a promising future for these next-generation types of intracortical electrodes in animal models.

## Data Availability Statement

The original contributions presented in the study are included in the article/Supplementary Material, further inquiries can be directed to the corresponding author/s.

## Ethics Statement

This study was approved and monitored by the Massachusetts General Hospital (MGH) Institutional Animal Care and Use Committee (IACUC).

## Author Contributions

AK conceived the study and experiments, fabricated the microdevice and injection tool, collected the data, and drafted the manuscript and figures. JW-W and CF aided with MRI data collection. SC provided the resources and supervision and helped to prepare the manuscript. All authors contributed to the article and approved the submitted version.

## Conflict of Interest

The authors declare that the research was conducted in the absence of any commercial or financial relationships that could be construed as a potential conflict of interest.

## Publisher’s Note

All claims expressed in this article are solely those of the authors and do not necessarily represent those of their affiliated organizations, or those of the publisher, the editors and the reviewers. Any product that may be evaluated in this article, or claim that may be made by its manufacturer, is not guaranteed or endorsed by the publisher.

## Supplementary Material

Supplementary Figure 1 Table showing the number of microdevices injected for each animal and their location in the brain.

Supplementary Figure 2 Tracking microdevice migration in the rodent brain using a 9.4T MRI scanner. (*Top row*) Two close-up axial MR images of the rat (#4) brain taken at different post-injection times, one MR image created by superimposing one over the other, and one created by subtraction. Microdevice D2 was injected (encircled in yellow) into M1. (*Bottom row*) Two close-up axial MR images of the rat (#5) brain taken at different post-injection times: one MR image created by superimposing one over the other and one created by subtraction. Microdevice D1 was injected (encircled in yellow) into the corpus callosum.

Supplementary Figure 3 Picture of a tissue block following brain fixation in PFA and trimming. The injected microdevice is encircled in yellow.

Supplementary Figure 4 Examples of unsuccessful MR images of the rat brain affected by (left) gradient duty cycle issues and (right) motion artifact.

Supplementary Figure 5 GFAP immunoreactivity to the microdevice (left) and microelectrode (right). Tissue slices were taken from rat #5 at 17 weeks post-injection/implantation. The plots show the average fluorescence pixel intensity of GFAP as a function of distance from the edge of the microdevice or microelectrode. The blue dashed line represents the full width at half maximum (FWHM).
