# Supplementary figures and images for: Tracking the Migration of Injectable Microdevices in the Rodent Brain Using a 9.4T Magnetic Resonance Imaging Scanner
Source: Front Neurosci. 2021 Oct 5;15:738589. doi: 10.3389/fnins.2021.738589 (PMC8524135; doi:10.3389/fnins.2021.738589)

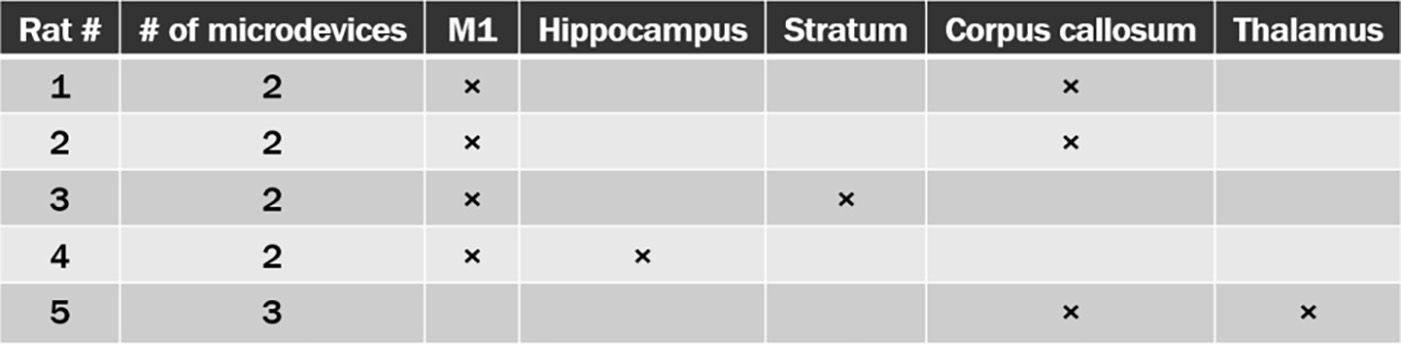

Supplement: Supplementary Figure 1 — Table showing the number of microdevices injected for each animal and their location in the brain. [file Image_1.jpg]

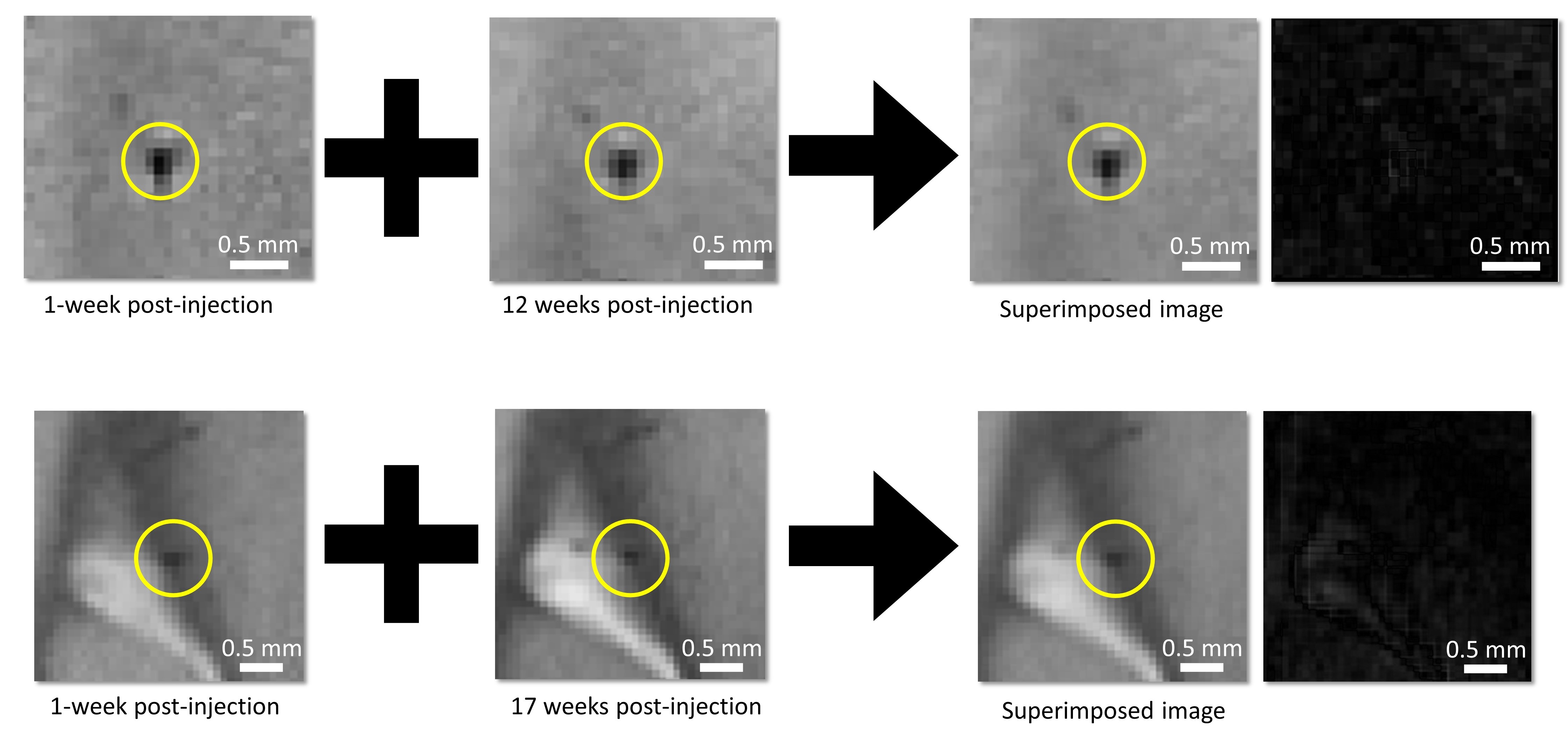

Supplement: Supplementary Figure 2 — Tracking microdevice migration in the rodent brain using a 9.4T MRI scanner. (Top row) Two close-up axial MR images of the rat (#4) brain taken at different post-injection times, one MR image created by superimposing one over the other, and one created by subtraction. Microdevice D2 was injected (encircled in yellow) into M1. (Bottom row) Two close-up axial MR images of the rat (#5) brain taken at different post-injection times: one MR image created by superimposing one over the other and one created by subtraction. Microdevice D1 was injected (encircled in yellow) into the corpus callosum. [file Image_2.JPEG]

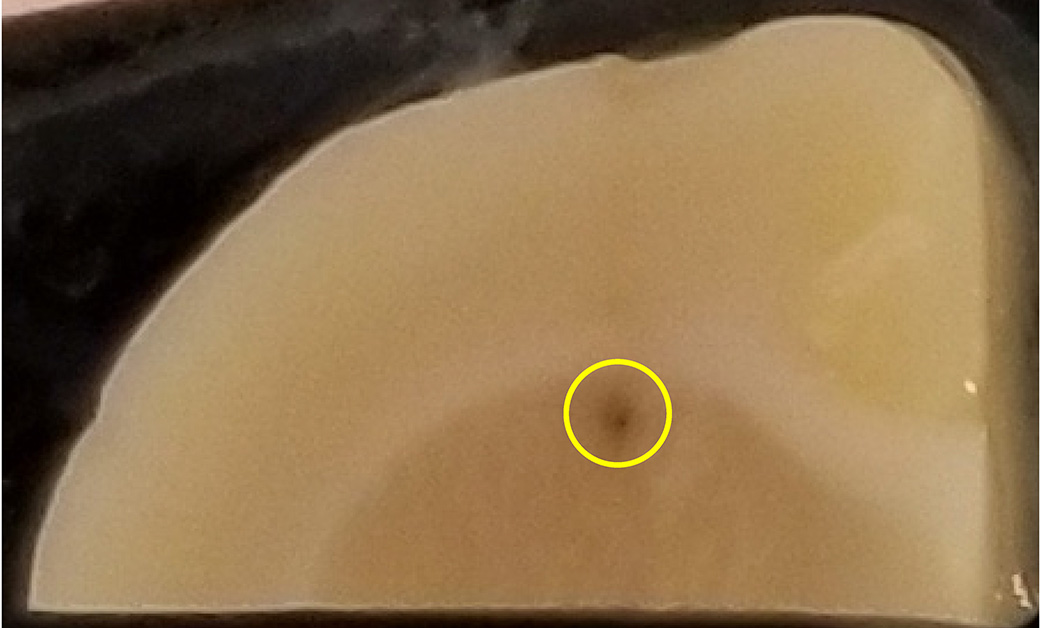

Supplement: Supplementary Figure 3 — Picture of a tissue block following brain fixation in PFA and trimming. The injected microdevice is encircled in yellow. [file Image_3.JPEG]

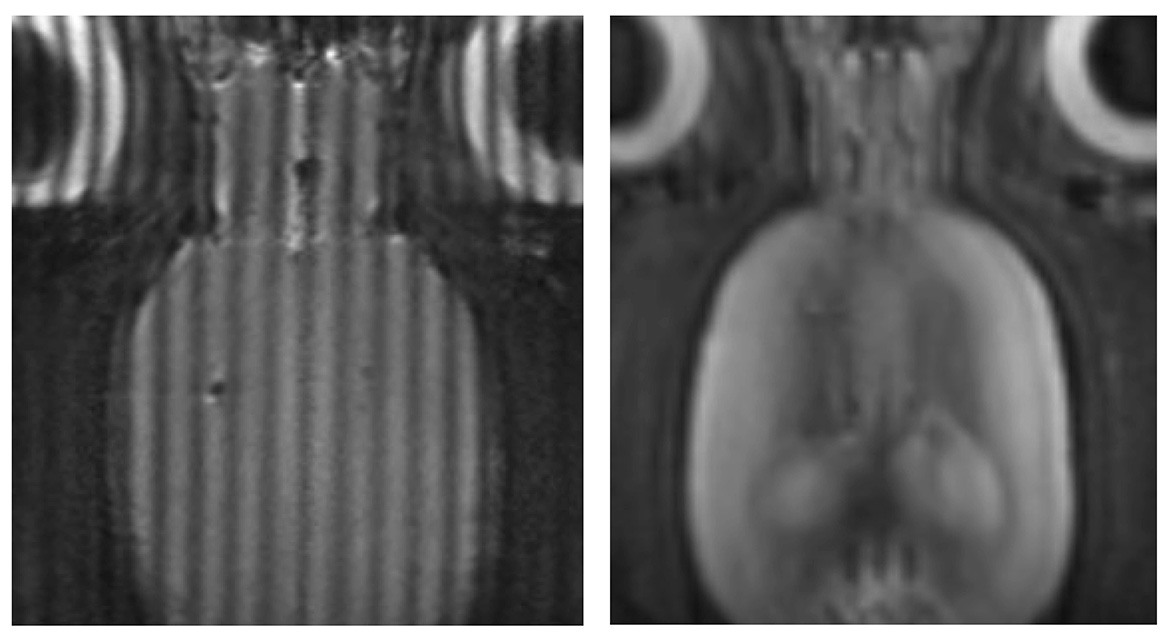

Supplement: Supplementary Figure 4 — Examples of unsuccessful MR images of the rat brain affected by (left) gradient duty cycle issues and (right) motion artifact. [file Image_4.JPEG]

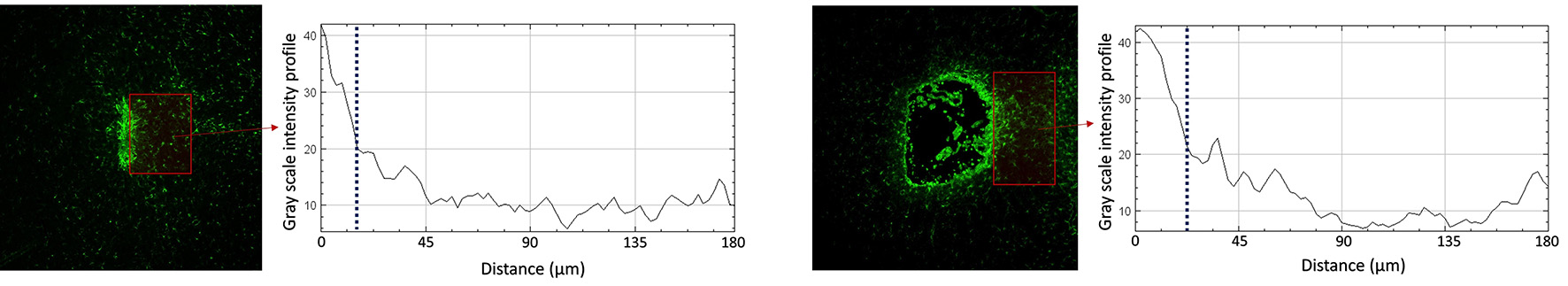

Supplement: Supplementary Figure 5 — GFAP immunoreactivity to the microdevice (left) and microelectrode (right). Tissue slices were taken from rat #5 at 17 weeks post-injection/implantation. The plots show the average fluorescence pixel intensity of GFAP as a function of distance from the edge of the microdevice or microelectrode. The blue dashed line represents the full width at half maximum (FWHM). [file Image_5.JPEG]
